# Proteome analysis of biomarkers in the cerebrospinal fluid of neuromyelitis optica patients

**Published:** 2009-08-19

**Authors:** Shumei Bai, Shilian Liu, Xuxiao Guo, Zhaoyu Qin, Banqin Wang, Xiaohong Li, Yanjiang Qin, Yi-Hsin Liu

**Affiliations:** 1 Institute of Biochemistry and Molecular Biology, School of Medicine, Shandong University, Shandong, People’s Republic of China; 2 Department of Clinical Laboratory, the Affiliated Hospital of Shandong University of Traditional Chinese Medical, Shandong, People’s Republic of China; 3 Biology Science Department in Marine College, Shandong University at Weihai, Shandong, People’s Republic of China; 4 Department of Neurology, Qilu Hospital of Shandong University, Shandong, People’s Republic of China; 5 Department of Ophthalmology, Keck School of Medicine, University of Southern California, Los Angeles, CA

## Abstract

**Purpose:**

To better understand the pathophysiological mechanisms underlying neuromyelitis optica (NMO), we developed a proteomics platform for biomarker discovery in the cerebrospinal fluid (CSF) of patients with NMO.

**Methods:**

Two-dimensional electrophoresis (2-DE) and matrix-assisted laser desorption ionization time of flight mass spectrometry (MALDI-TOF MS) were used to compare the CSF proteome of NMO patients with that of controls. A subsequent ELISA and western blot analysis were performed to verify the results of the proteomic analysis. Pathway Studio 5.0 software was used to determine possible functional interactions among these differentially expressed proteins.

**Results:**

Using 2-DE and MALDI-TOF MS, we identified 11 differentially expressed proteins and two isoforms of these same proteins. The expression of four proteins was enhanced, whereas the expression of seven proteins was reduced in the NMO group in comparison to the control group. These differences in protein expression were confirmed by performing ELISA and western blot analyses (p<0.01). Protein network analyses revealed biologic interactions and cross-talks among these differentially expressed proteins.

**Conclusions:**

Because of their unique expression profile in NMO CSFs, these proteins are candidate biomarkers for NMO. Thus, our findings may have important implications for both the diagnosis of NMO and the further understanding of its pathogenesis.

## Introduction

Neuromyelitis optica (NMO), also termed Devic's syndrome, is an idiopathic inflammatory demyelinating disease of the central nervous system (CNS) predominantly affecting optic nerves and the spinal cord [1]. It is a disabling and, occasionally, life-threatening disease. Patients with NMO can experience relapses of optic neuritis or myelitis that occur in intervals of months or years. NMO is considered to be a severe monophasic syndrome characterized by bilateral optic neuritis (ON) and myelitis occurring in rapid succession. Furthermore, the prognosis for patients with NMO is often poor; within five years of disease onset, more than half of patients will develop severe bilateral visual impairment and even visual loss in at least one eye and /or inability to ambulate without assistance within 5 years of disease onset [2-4]. Serum NMO-immunoglobulin G (IgG) has been investigated, and its presence is highly specific for NMO [5,6]. However, NMO-IgG is not detectable in all patients. Therefore, the diagnosis of NMO is generally based on the combination of clinical, neuroimaging, laboratory, and pathological findings [7,8]. It is thus crucial to develop diagnostic tools for NMO. Abnormal cerebrospinal fluid (CSF) findings are one of the characteristics of NMO and may provide important information for deciphering mechanisms of NMO pathogenesis. It is well accepted that CSF proteomics is a powerful technique for monitoring protein expression in the CSF [9]. Moreover, it can provide valuable information about the CNS disease in general [10,11]. In this study, we examined the CSF proteome profile of NMO by means of proteomic analysis with two-dimensional electrophoresis (2-DE), followed by matrix-assisted laser desorption ionization time of flight mass spectrometry (MALDI-TOF MS) and database searching. We confirmed our findings with ELISA, western blot analyses, and the establishment of the protein network. Understanding the expression and function of these disease-related proteins may enable us to identify them as candidate biomarkers to aid in the diagnosis of NMO and, perhaps, in its treatment and prognosis.

## Methods

### CSF samples

We collected CSF samples from 34 NMO patients and 39 controls from Qilu Hospital of Shandong University, Jinan, China. CSF samples collected from nine NMO patients (six females and three males; age: 38.1±4.8 years old) and nine controls (five females and four males; age: 36.5±6.4 years old) were used to perform 2-DE. CSF samples collected from 25 NMO patients (16 females and nine males; age: 35.4±4.2 years old) and 30 controls (15 females and 15 males; age: 38.6±6.5 years old) were used for ELISA. CSF samples from 15 NMO patients (10 females and five males; age: 37.5±5.3 years old) and 15 controls (eight females and seven males; age: 35.2±4.6 years old) from the ELISA analysis were selected at random for western blot analysis. Patients with NMO enrolled in this study received their diagnosis based on the criteria of Wingerchuck et al. [12]. The criteria of the NMO: Patients with a monophasic or relapsing course of NMO; the cells (polymorphonuclear) >50 /mm^3^ in CSF of NMO patients; the lesions extend over three or more vertebral segments on spinal cord MRI. We collected CSF from NMO patients whose clinical signs persisted for more than five days. The clinical signs of NMO patients includes the abnormal signs of the CSF, the MRI and the other abnormal signs in the nervous system. Examination of the CSF was performed when clinical symptoms were apparent on average for 5 days. CSF from NMO patients showed average cell densities of 52.5±4.3/mm^3^ and 2–5 oligoclonal bands (OGB). Whereas in control samples, MRI showed normal vertebral columns. CSF cell counts were 5.0±1.8/mm3 and OGBs were not detectable in the CSF. Spinal cord MRI showed hyperintensive signal (HS) greater than three vertebral segments (HS>3 in T2-weighted MRI). The MRIs were performed for the whole patients (NMO patients and the controls). Whereas in control samples CSF cell counts were 5.0±1.8/mm^3^ and OGBs were not detectable in the CSF. MRI showed normal vertebral columns. While first-line therapy for acute attacks of NMO is intravenous corticosteroids, CSF samples were collected from patients before initiating corticosteroid or other immunosuppressive therapy to avoid affecting CSF results. Among control patients (n=6 with tension-type headache, n=1 each with drug-induced delirium, normal pressure hydrocephalus, and trigeminal neuralgia), the diagnosis was defined according to individual disease criteria. The CSF was analyzed to rule out encephalitis and subarachnoid hemorrhage, and routine work-up revealed normal clinical and imaging results. The clinical features of the study participants are summarized in Table 1.

**Table 1:** Clinical features and laboratory values of NMO patients and controls.

**Diagnosis**	**Number (female/male)**	**Age (years)**	**CSF cells** **(/mm3)**	**OGBs^a^ (bands)**	**Spinal cord MRI**	**Disease duration (days)**
NMO	9 (6/3)	38.1±4.8	52.5±4.3	2–5	HS^b^>3 vertebral segments	5 (3–7)
Controls	9 (5/4)	36.5±6.4	5.0±1.8	0	–	4 (2–6)

Abbreviations: ^a^OGBs represents oligoclonal band, ^b^HS represents hyperintensive signal in T2-weighted MRI.

This study was approved by the Human Ethics Committee of Shandong Province, China. Written informed consent was obtained from each patient. All CSF samples used in this study were obtained from patients undergoing a lumbar puncture in the L4–L5 vertebral interspace. Each patient had 2 mL of CSF removed. CSF samples were centrifuged at 1,000× g (4 °C) for 10 min to eliminate cells and other insoluble material. The samples were then stored at −80 °C for further study.

### 2-DE

To display total protein content in the CSF, high-abundance proteins, such as IgG and albumin, were not depleted. Initially, 500 μl CSF was mixed with 2,000 μl of ice-cold acetone and stored overnight at −20 °C to remove salt from each sample [13]. After centrifugation at 16,000× g at 4 °C for 30 min, the protein pellet was washed twice with 90% acetone and air-dried. Then, precipitated proteins were solubilized in lysis buffer containing 7 M urea, 2 M thiourea, 4% cholamidopropyl dimethylamino propanesulfonate (CHAPS), 65 mM dithiothreitol (DTT), and 2% immobilized pH gradient (IPG), pH 3–10 nonlinear (NL; GE Healthcare, Piscataway, NJ) for 30 min at room temperature. A Bradford assay was performed to determine the protein concentration [14]. The IPGphor system (GE Healthcare) with 18 cm IPG strip was used for isoelectric focusing electrophoresis (IEF) at pH 3–10 NL. Next, 100 μg of the protein for analytical runs or 1,000 μg of the protein for preparative runs were mixed with the rehydration solution containing 8 M urea, 2% CHAPS, 18 mM DTT, and 0.5% IPG buffer to obtain a total volume of 340 μl per strip. After rehydration for 12 h at 20 °C, IEF was performed as follows: 200 V for 1 h, 500 V for 1 h, 1,000 V for 1 h, gradient to 8,000 V over 2 h, 8,000 V for 60,000 V hours. Vertical SDS–PAGE (12.5% T, 2.75% C) was run in a Protean II xi two-dimensional cell (Bio-Rad, Hercules, CA). The preparative gels were stained with Coomassie brilliant blue G-250, and the analytical gels were stained with silver according to manufacturer’s instructions.

### Image analysis

Silver-stained gels were scanned using an UMAX PowerLook 2100XL Image Scanner (GE Healthcare) with standard conditions for all gels. Intensity calibration was performed using an intensity step wedge (detection parameters: Smooth 4; Min Area 10; Saliency 1.5). Image spots were initially detected, matched, and manually edited with the ImageMaster™ 2D Platinum 5.0 software (GE Healthcare). Quantification was given as a spot volume percentage (vol %), with each single spot volume normalized with respect to the total spot volume of the 2-DE gel. The differences in spot volume was used as a measure of changes in protein expression levels between the NMO and control groups, and were ascertained statistically by the Mann–Whitney *U*-test, where a p value <0.05 was considered significant. The *M*_r_ and pI of each protein spot were calibrated against known protein markers.

### MALDI-TOF MS and database searching

Differentially expressed protein spots were manually excised from the preparative gels. Excised gel pieces were destained in 50 mM NH_4_HCO_3_/50% acetonitrile (ACN) to remove all traces of Coomassie brilliant blue. Destained gel pieces were dried by vacuum centrifugation and resuspended in 50 mM NH_4_HCO_3_ solution containing 10 ng/μl trypsin. Trypsin digestion was performed at 37 °C for 24 h. The resulting peptide mixtures were extracted with 200 μl of 60% ACN and 0.1% trifluoroacetic acid (TFA) for 20 min with sonication, and the supernatant was removed. The peptide mixtures were deposited on the MALDI probe (the matrix were 2 mg/ml α-cyano4-hydroxy cinnamic acid in 50% acetonitrile, 0.1% trifluoroacetic acid containing 10 mM ammonium phosphate) and analyzed on a MALDI-TOF 4700 (ABI Corp, Framingham, MA). The MALDI/TOF mass spectra were recorded in positive ion reflection mode. The accelerating potential was 20 kV, and the trypsin autodigestion peaks were used as external calibration. When we analyzed the spectrum peaks, the trypsin autodigestion peaks were filtered. Peptide mass fingerprints (PMF) were obtained after processeing by Data Explorer software softwareVersion 4.0.0.0 (GE Healthcare, Piscataway, NJ). The peptide mass data were searched against Swiss-Prot protein database with the Mascot search engine and with *Homo sapiens* as the species searched.

### ELISA analysis of haptoglobin

CSF samples from 25 NMO patients (35.4±4.2 years of age) and 30 controls (38.6±6.5 years of age) were used for the ELISA test. The samples for ELISA analysis were selected from the 34 NMO patients and 39 controls at random. An ELISA kit for human haptoglobin (HP; Gentaur, Guangzhou, China) was used, and the assays were run according to manufacturer’s directions. The data were acquired on a model 680 microplate reader (Bio-Rad) with the absorbance wavelength set at 450 nm.

### Western blot analysis

Semiquantitation of vitamin D-binding protein (DBP) was conducted by performing western blot analysis. CSF samples from 15 NMO (37.5±5.3 years of age) patients and 15 controls (35.2±4.6 years of age) were used for this study. Each CSF sample was loaded twice onto the PAGE gel. Briefly, 8 μg of CSF protein was mixed with 4× loading buffer containing 0.5 M Tris-HCl (pH 6.8), 8% SDS, 0.4% Bromophenol Blue, 40% glycerin and 5% β-Mercaptoethanol. The mixture was boiled for 10 min. 1-DE separation was performed on a 10% SDS polyacryamide gel, and 8 μg of CSF protein were loaded per lane with loading buffer. Next, 5 μl of Easy See Western Marker (TransGen Biotech Co., Ltd, Beijing, China) was loaded onto the first lane. Proteins were blotted onto the polyvinylidene difluoride (PVDF) membrane [15]. When the transfer electrophoresis was completed, the gel was stained with Coomassie Blue R250 to confirm whether the proteins had transferred completely from the gel to the PVDF membrane. Mouse monoclonal anti-vitamin D binding protein (DBP; HYB 249–01; Abcam, Cambridge, MA) was used to probe DBP, and a peroxidase-conjugated goat anti-mouse IgG (ZSGB-Bio, Guangzhou, China) was used for detection. The primary antibody was HYB 249–01 mouse monoclonal anti-(human) vitamin D binding protein. The color reaction was developed using the Enhanced Chemiluminescence Plus System (Amersham Biosciences, Piscataway, NJ). Protein bands were digitally scanned, and the densities of each individual band were analyzed using Image Quant TL software version 2005 (Amersham Biosciences).

### Protein network analyses with Pathway Studio 5.0

To construct a protein interaction map, we used the Pathway Studio 5.0 software (Ariadne Genomics, Rockville, MD). It is able to dynamically create and draw protein interaction networks and pathways. Each node represents either a protein entity or a control mechanism of the interaction. It contains pathway components, protein–protein interactions, proteins and their cellular processes, small molecules, functional classes, and treatments. In addition, it has several path analyses and databases. In this study, we selected the shortest path analysis and the ResNet Databases.

### Data analysis

Results were expressed as mean±standard deviation (SD). Differences in protein spots (volume %) between the control group and the NMO group were subjected to the Mann–Whitney *U*-test. The Student *t-*test was applied to data collected by ELISA and western blot analysis. A p value < 0.05 was considered significant.

## Results

### 2-DE patterns and identification of differentially expressed proteins in the NMO CSF

**Figure 1:**
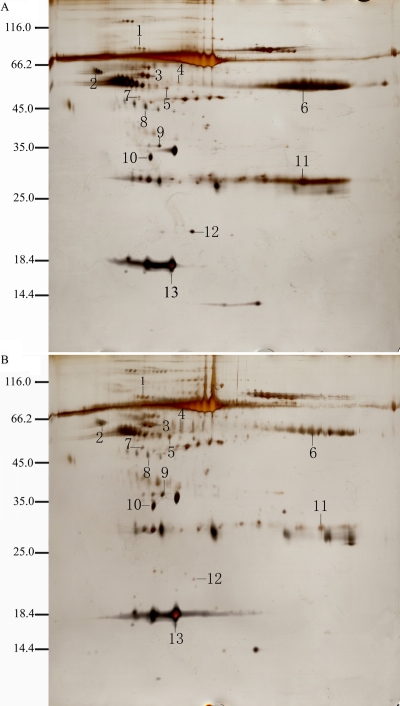
2-DE results. A representative two-dimensional electrophoresis (2-DE) gel images of cerebrospinal fluid proteins of neuromyelitis optica (NMO; **A**) and control (**B**). Numbers indicate proteins that were expressed differentially in the two groups. Protein spots 2, 6, 8, 11, and 12 were upregulated, whereas the other spots were downregulated in the NMO group (**A**) compared to the control group (**B**).

**Figure 2:**
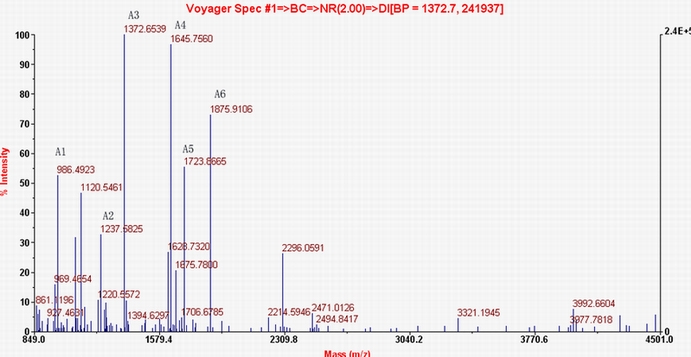
Peptide mass fingerprint spectrum of alpha-1β-glycoprotein. The x-axis represents the mass-to-charge ratio (m/z), and the y-axis represents the relative abundance. A1-A6 show the identified peptide fragments (A1: CLAPLEGAR; A2: LETPDFQLFK; A3: HQFLLTGDTQGR; A4: CEGPIPDVTFELLR; A5: LELHVDGPPPRPQLR; A6: VTLTCVAPLSGVDFQLR).

CSF proteins taken from both the experimental (n=9) and control groups (n=9) were separated by 2-DE. Two representative silver stained 2-DE gels, which corresponded to proteomic profiles of the NMO group or the control group, respectively, are presented in Figure 1A,B . With the help of ImageMaster™ 2D Platinum 5.0 software, we detected 388±27 protein spots in the NMO samples and 405±19 protein spots in the control samples. A total of 42 distinct protein spots were detected in the CSF from the NMO patients according to ImageMaster™ 2D Platinum 5.0 software analysis. These differentially expressed protein spots were extracted from Coomassie brilliant blue-stained gels and analyzed by MALDI-TOF MS. Raw data from the MS were used to search Swissprot database with the Mascot search engine (Appendix 1). Neurofilament (NF), HP, immunoglobulin kappa chain C region (IGKC), immunoglobulin heavy chain gamma 3 (IGHG3) were upregulated; whereas alpha-1β-glycoprotein (A1BG), DBP, fibrinogen gamma chain (FGG), pigment epithelium-derived factor (PEDF), apolipoprotein A-IV (ApoA-IV), apolipoprotein E (ApoE), and transthyretin (TTR) were downregulated in the NMO group in comparison to the control group. Spot 1 was identified as A1BG with a summary score of 96 and a protein coverage of 54.3%. However, the second-best match of this spot only had a summary score of 22 and a protein coverage of 9.6%. Figure 2 shows the PMF and matched peptide fragments (A1-A6) of A1BG. The MW in kDa, pI, volume%, *p*-value, and MS results are presented in Table 2. Some of these protein spots were identified as the same protein (such as spots 8 and 12) by MALDI-TOF-MS. Protein degradation and fragmentation of proteins, or post-translational modifications, such as phosphorylation, or different isoforms of the same proteins may explain this phenomenon.

**Table 2:** Informatics of Proteins and their isoforms acquired by the 2-DE and MS^a^.

**Number**	**Protein identified**	**Swiss-prot**	**Accession number**	**Experimental (MW/pI)**	**Volume%**		**p-value**	**Sequence coverage (%)**	**Score**
NMO	Control
1	alpha-1β-glycoprotein	A1BG_HUMAN	P04217	66.42/5.32	0.085±0.028	0.166±0.087	0.027	54.3	96
2	Neurofilament	NFL_HUMAN	P07196	55.36/5.05	0.138±0.040	0.083±0.020	0.001	9	55
3	vitamin D-binding protein	VTDB_HUMAN	P02774	53.65/5.34	0.221±0.101	0.348±0.101	0.03	56.1	242
4	fibrinogen gamma chain	FIBG_HUMAN	P02679	52.25/5.82	0.062±0.019	0.113±0.028	0.003	12.2	88
5	pigment epithelium-derived factor	PEDF_HUMAN	P36955	50.02/5.75	0.106±0.038	0.215±0.098	0.014	40.1	162
6	immunoglobulin heavy chain gamma 3	IGHG3_HUMAN	P01860	52.46/8.10	2.943±0.462	1.767±0.467	0.0002	23.4	139
7	apolipoprotein A-IV	APOA4_HUMAN	P06727	44.23/5.25	0.028±0.010	0.064±0.012	0.0001	52.1	265
8	Haptoglobin	HP_HUMAN	P00738	44.21/5.35	0.101±0.037	0.067±0.023	0.038	19.1	79
9	apolipoprotein E	APOE_HUMAN	P02649	34.25/5.45	0.156±0.054	0.264±0.107	0.01	41.9	181
10	Transthyretin	TTHY_HUMAN	P02766	33.00/5.40	0.293±0.082	0.462±0.097	0.001	63.3	135
11	Ig kappa chain C region	KAC_HUMAN	P01834	27.00/7.50	2.212±0.570	1.111±0.209	0.027	43.6	66
12	Haptoglobin	HP_HUMAN	P00738	22.90/6.00	0.270±0.09	0.102±0.05	0.0008	29.4	82
13	Transthyretin	TTHY_HUMAN	P02766	16.58/5.54	1.564±0.563	2.700±0.516	0.0008	59.7	155

Abbreviations: ^a^No represents number of protein spots; Acc. no represents identification of proteins indicated as Swiss-prot accession numbers; p value obtained by Mann–Whitney U-test.

151515151515

### Verification of HP by ELISA

The HP level in the NMO group (n=25) was 2.67±0.92 μg/mL. This was higher than that of the control group (n=30), which exhibited a concentration of 2.08±0.76 μg/ml (p<0.05). HP levels >2.20 μg/ml consistently identified patients with NMO with 92.0% sensitivity and 83.3% specificity. The Sensitivity was the percentage of the NMO patients whose HP levels > 2.20 μg/ml account for the whole NMO patients. The Specificity was the percentage of the controls whose HP levels < 2.20 μg/ml account for the whole controls. The Accuracy rate was the percentage of the NMO patients whose HP levels > 2.20 μg/ml and the controls whose HP levels < 2.20 μg/ml account for the whole participants.The accuracy rate was 87.3%. Thus, HP ELISA may become an excellent screening tool for the diagnosis of NMO.

### Verification of DBP by western blot

**Figure 3:**
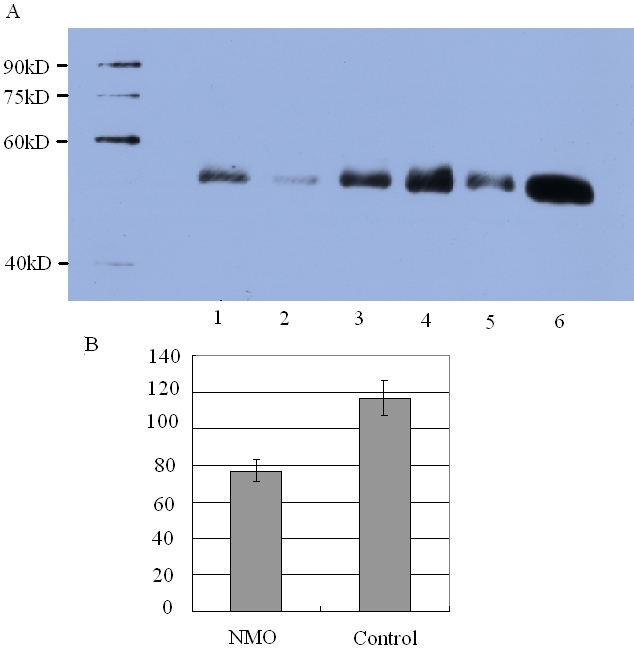
Western-blot results. **A:** A typical western blot image against vitamin D-binding protein (DBP) in CSF of 15 NMO patients: 1–3 were from NMO patients and 4–6 were from controls. **B:** Densitometric analysis of DBP bands in the two groups. Each column represents the mean±standard deviation (n=15). The average density value of the NMO group was 77±3.87, and the average density value of the control group was 117±5.45 (p<0.01). DBP level was reduced in NMO by 34% as compared to control samples

Samples from 15 NMO patients and 15 controls were selected for western blot analysis. The results presented in Figure 3A confirmed DBP as a differentially expressed target, as seen in the 2-DE experiments. Semiquantitative densitometric evaluation of the detected protein bands revealed that DBP levels were decreased in the NMO group by 34%. The average band intensity in the NMO group was 77±3.87, while the average band intensity in the control group was 117±5.45 (p<0.01). A typical western-blot image is shown in Figure 3B.

### Protein network

**Figure 4:**
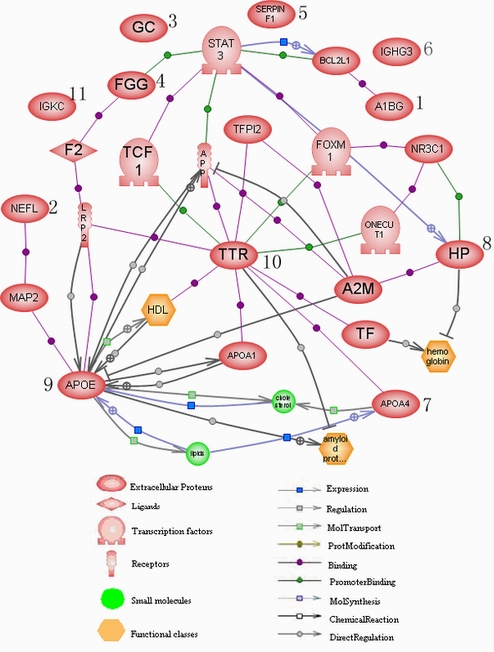
Protein network of the identified proteins constructed with the PathwayStudio 5.0 software. Each node represents either a protein entity or a control mechanism of the interaction. The legend of the interaction network is summarized under the figure. Numbers show the identified proteins: alpha-1β-glycoprotein (A1BG), neurofilament (NF), vitamin D-binding protein (DBP), fibrinogen gamma chain (FGG), pigment epithelium-derived factor (PEDF), immunoglobulin heavy chain gamma 3 (IGHG3), apolipoprotein A-IV (ApoA-IV), haptoglobin (HP), apolipoprotein E (ApoE), transthyretin (TTR), and immunoglobulin kappa chain C region (IGKC). These proteins are related to some functional classes and small molecules. The relations of binding, expression, and direct regulation were obviously displayed among these proteins.

To determine possible functional cross-talk among differentially expressed target proteins, we built a protein network with Pathway Studio 5.0 software (Figure 4). With multiple interacting binding partners, transthyretin (TTR) functions as an anchoring factor that pulls together possible direct or indirect interactions among HP, ApoE, ApoA-IV, FGG, A1BG, NF, and their transcriptional regulators.

## Discussion

To our knowledge, this is the first comprehensive proteomic study profiling differential protein expression in the CSF of NMO patients. There have been few studies characterizing serum proteins or CSF proteins in NMO patients using immunological techniques or ELISA assay [16-18]. In a previous study, 2-DE profiling of phosphotyrosyl proteins in human CSF identified 500 protein spots [9,10]. In the current study, we identified 11 proteins and 2 isoforms that were differentially expressed in the CSF of NMO patients compared with the controls. Many of these identified proteins have more than one function, and they fall under four groups based on their biochemical characteristics and functions. These proteins can be classified based on their pro or anti- inflammatory functions, binding or transport functions, or as apolipoproteins, and proteins involved in neurologic disorders.

### Proinflammatory or antiinflammatory proteins

Several investigators have analyzed the CSF proteome in patients with neurologic diseases [19-22]. In most of those studies, high-abundance proteins, such as IgG and albumin, were removed to enhance the detection of low-abundance proteins. However, such a treatment also results in the loss of some low-abundance proteins. In our current study, CSF proteins were profiled in their entirety so our results provided a more detailed look at the proteins that are normally present in the CSF. Hence, we were able to better show the changes in protein expression that had occurred due to pathological conditions of NMO.

Notably, the significant increase in the levels of IGHG3 and IGKC in the CSF of NMO patients suggest that immunological mechanisms are involved in the pathophysiology of NMO [23]. An intrathecal IgG synthesis inherent to NMO and a compromised blood-brain barrier are possible mechanisms that can lead to higher levels of immunoglobulin [24].

HP is an acute phase protein that binds free hemoglobin and exerts antioxidant properties [25]. HP has been proposed to be involved in a highly interactive ensemble of lymphocytes, neutrophils, and monocytes participating in inflammatory processes, and it has been observed in conditions of extensive tissue damage and necrosis [26,27]. In this study, we found a high level of HP in the CSF of patients with NMO. Subsequent ELISA confirmed this finding. Moreover, there were reports showing elevated HP levels in the CSF of patients with Guillain-Barré syndrome, a peripheral neuropathy that causes acute neuromuscular failure [19,20]. Thus, recognizing and monitoring changes in HP may reveal useful information regarding the disease process and yield predictors for various disease parameters in NMO. Further follow-up studies on HP in the CSF of NMO patients are warranted.

Alpha-1-B glycoprotein (A1BG) is a plasma protein of unknown function. Conservation at the sequence level with immuoglobulins and other members of the immunoglobulin supergene family suggests its possible function in the immune system and in cell adhesion [28]. A1BG physically interacts with BCL2L1 and its activities may be modulated by a signal transducer and activator of transcription 3 (STAT3) through BCL2L1 (Figure 4). It had been reported that STAT3 might regulate the transcription of anti-apoptotic proteins such as BCL-XL that control cell growth and survival [29,30]. Thus, A1BG and BCL2L1 may play a role in the pathogenesis of NMO and may become potential therapeutic targets.

Fibrinogen gamma (FGG) is one of the target genes of STAT3, as STAT3 is reported to control the transcription of the FGG gene [31]. FGG is a plasma glycoprotein with functions in wound healing and anti-inflammation [32]. Tissue injury in the CNS has been correlated with a post-traumatic local increase in fibrinolysis as well as to an inflammatory response [33]. Together, these proteins directly or indirectly participate in the inflammatory state of NMO, which may offer new opportunities for biomarker discovery and the study of the pathogenesis of NMO.

### Binding or transport protein

DBP, also known as group-specific component globulin (Gc-globulin), is synthesized predominantly by hepatic parenchymal cells [34]. DBP is a plasma protein with many important functions. It acts as a carrier protein for vitamin D and its plasma metabolites, and it also serves as a scavenger protein to clear endotoxins and extracellular G-actin that are released from necrotic cells [35]. DBP has other potential roles, such as responding to acute tissue injury through conversion to a macrophage-activating factor, neutrophil chemotactic activity, and enhancement of C5a-mediated signaling [36]. Geographic, biologic, and immunologic evidence supports the relationship between low environmental supplies of vitamin D and an increased risk of developing multiple sclerosis (MS) [37]. MS has a typical geographical distribution with a low prevalence in equatorial regions and an increasing prevalence with increasing latitudes in both hemispheres. For example, in the northern regions of Europe and the USA an increased MS prevalence is shown in comparison with the southern regions. Whereas in Australia, an increased prevalence is seen in the southern coast in comparison with the sub-tropical northern coast. Whereas in Australia, there has been an increased prevalence in the southern coast as compared to the subtropical northern coast. The DBP level is reportedly decreased in the CSF of MS patients [38]. In our current study, 2-DE and western blot analysis revealed significantly reduced levels of DBP in the CSF samples from the NMO group.

TTR is a beta-sheet rich protein that is synthesized predominantly by parenchymal cells of the liver. It may cleave the C-terminus of ApoA-I, reducing its capacity to promote high density lipoprotein (HDL)-mediated cholesterol efflux and increasing ApoA-I amyloidogenicity [39]. As shown in Figure 4, TTR, ApoA-I, and HDL strictly interact through physical binding. TTR is a known negative acute phase protein that is significantly reduced in the CSF of amyotrophic lateral sclerosis patients [40]. We also found that TTR was decreased in the CSF of patients with NMO.

### Apolipoproteins

ApoA-IV is synthesized primarily in the intestine and secreted into the plasma. Acute inflammation can affect plasma ApoA-IV levels [41]. The decreased level of ApoA-IV in the CSF of NMO patients may reflect the inflammation status of NMO. Moreover, Langner reported that ApoA-IV was associated with myelin biosynthesis [42].

ApoE has multiple biologic properties aside from its physiologic role in cholesterol transport. ApoE has immunomodulatory properties both in vitro and in vivo [43]. It directly regulates ApoA-1, HDL, and amyloid precursor protein (APP; Figure 4). ApoE is reported to have neurotrophic activity, and CSF ApoE levels are reduced in MS, a disease analogous to NMO [44]. Therefore, reduced levels of ApoE may contribute to the progression of NMO.

### Expression proteins related to neurologic disorders

NFs are predominantly localized in axons, and their relative abundance reflects the degree of compromised axonal function or axonal degeneration [45]. Miyazawa and associates reported finding high concentrations of NF heavy chains in the CSF of NMO patients [16]. The raised level of NF suggests its potential value as a marker of axonal damage and neuronal degeneration in NMO.

Pigment epithelium-derived factor (PEDF), also called SERPINF1, is a neuroprotective and anti-angiogenic factor [46] that is widely expressed in the CNS, including the retina, and in most tissues of the body. PEDF is an important neuromodulatory factor that can provide effective neuroprotection and a primary endogenous angiogenic inhibitor in the eye [47]. Trauma or lesion to the optic nerve may influence the level of PEDF.

### Conclusion

From our study, which used proteomic approaches, we have presented the finding of proteins differentially expressed in the CSF of NMO patients. Two candidate proteins, HP and DBP, whose expression levels were either elevated or reduced under the NMO pathological condition, were confirmed by ELISA and western blot analysis, respectively. Recognition and characterization of these proteins and their alterations will likely yield a rich source of information for describing the disease process and for helping to reveal predictors of various disease parameters in NMO. Although some of these proteins are not specifically affected in different inflammatory neurologic diseases and may be of limited value as disease-related biomarkers, these proteins may serve as candidate biomarkers for NMO when examined in combination. Further steps with a larger group of patients with NMO, including different subtypes such as optic neuritis and myelitis, will substantiate these findings.

## Appendix 1. Peptide sequence data.

To access the data, click or select the words “Appendix 1.” This will initiate the download of a pdf archive of the file. Peptide sequence data acquired by MALDI-TOF MS were searched against the Mascot database to obtain best matches to known proteins in the SWISS-PROT Database.
